# Leucorrhœa

**Published:** 1854-12

**Authors:** 


Leucorrhœa.—Dr. Mayer recommends that in the treatment of leucorrhœa, cylinders formed of fine muslin and "charpie" should be soaked in a solution of alum, sulphate of zinc, of iron, or of nitrate of silver, and introduced into the vagina by means of the speculum, which is withdrawn when the cylinder has been introduced into it. The cylinder must be of good size, so that the folds of the vagina may be obliterated. The cylinder is retained in for ten or twelve hours. The alum solution is preferred by the author, and its strength is one part to fifty, to twenty-five, and to twelve of water successively. Three cases only are referred to, and all were in prostitutes. In the first case the cylinder was introduced six times; in the second, fifteen; in the third, five times.

